# Optimal beam sources for Stark decelerators in collision experiments: a tutorial review

**DOI:** 10.1140/epjti/s40485-015-0021-y

**Published:** 2015-08-06

**Authors:** Sjoerd N Vogels, Zhi Gao, Sebastiaan YT van de Meerakker

**Affiliations:** Radboud University, Institute for Molecules and Materials, Heijendaalseweg 135, AJ Nijmegen, 6525 Netherlands

**Keywords:** Sample, Article, Author

## Abstract

With the Stark deceleration technique, packets of molecules with a tunable velocity, a narrow velocity spread, and a high state purity can be produced. These tamed molecular beams find applications in high resolution spectroscopy, cold molecule trapping, and controlled scattering experiments. The quality and purity of the packets of molecules emerging from the decelerator critically depend on the specifications of the decelerator, but also on the characteristics of the molecular beam pulse with which the decelerator is loaded. We consider three frequently used molecular beam sources, and discuss their suitability for molecular beam deceleration experiments, in particular with the application in crossed beam scattering in mind. The performance of two valves in particular, the Nijmegen Pulsed Valve and the Jordan Valve, is illustrated by decelerating ND _3_ molecules in a 2.6 meter-long Stark decelerator. We describe a protocol to characterize the valve, and to optimally load the pulse of molecules into the decelerator. We characterize the valves regarding opening time duration, optimal valve-to-skimmer distance, mean velocity, velocity spread, state purity, and relative intensity.

## Introduction

Over the last decades, there has been tremendous progress in the ability to manipulate molecular beams. Nowadays, full control over both the internal and external degrees of freedom of molecules in a beam is possible. Efficient molecular beam sources are available to cool the rotational and vibrational degrees of freedom in a supersonic expansion, yielding beams with a high state purity and velocity distributions corresponding to temperatures of 1 K or less. Further manipulation of the beam has become possible since the advent of molecular beam decelerators [1–3]. Molecules can be decelerated or accelerated to any desired velocity exploiting the interaction of molecules with electric or magnetic fields, using techniques akin to the manipulation of charged particles in LINACs. These Stark and Zeeman decelerators offer the revolutionary capability to produce packets of molecules with a tunable velocity, a narrow velocity and angular spread, and almost perfect quantum state purity [4–8].

Since the first demonstration of a Stark decelerator in 1999 [1], various decelerators were built ranging from several-meter-long structures [9] to decelerators integrated on a chip [10, 11]. Distinct deceleration concepts were developed to optimize the efficiency of the deceleration process [2, 3, 9, 12–16]. Stark and Zeeman decelerators have been implemented in various laboratories, and successfully used in various applications ranging from ultra-high resolution spectroscopy [17, 18] and cold molecule trapping [19–22] to precise crossed beam scattering experiments [23–27].

A Stark or Zeeman decelerator selects a portion of the initial molecular beam pulse, and transfers this portion to the desired final velocity keeping the phase-space density in the selected bunch constant [28]. In this respect, the decelerator acts as a filter, in principle leaving the part of the beam that is not selected to propagate freely. The quality of the bunch of molecules exiting the decelerator therefore critically depends on the parameters of the initial molecular beam pulse and the pulsed valve used. Several molecular beam sources are used in laboratories around the world as a starting point for deceleration experiments. Some of them are home-built, others are commercially available. Frequently used valves include the piezo activated valve [29], General Valve, Jordan Valve, Even-Lavie Valve [30], and more recently the Nijmegen Pulsed Valve [31]. Their opening mechanisms and technical sophistication are equally diverse as their performance, availability and purchasing cost.

Although the working principles of decelerators are well documented in the literature, and the (dis)advantages of the various decelerator concepts are discussed [6], generation of the initial molecular beam pulse has received little attention. On the one hand this is surprising, as a proper choice and implementation of the source directly influences the quality of the overall experiment. On the other hand, this is not surprising, since a quantitative comparison of different valve performances is extremely difficult to make. The parameter space is vast, and depending on the application, different criteria exist for the bunch of molecules exiting the decelerator. For trapping experiments, for instance, the sole interest may be to achieve the highest possible phase-space density at low final velocities. For crossed beam scattering experiments, on the other hand, one wishes to access a wide range of final velocities, ranging from velocities below 100 m/s to velocities up to several hundreds m/s. This requires the use of various seed gases to produce the molecular beams, and the ability to select final velocities that are already present in the initial molecular beam pulse. The selected packet of molecules exiting the decelerator should be sufficiently decoupled from the remainder of the molecular beam pulse. This, in turn, imposes stringent requirements on the speed and temporal distribution of the initial beam pulse.

So which valve should one ideally use for a specific deceleration experiment? As a beginning PhD student, you may ask this question to different professors, and for the reasons given above, you will very likely get an equal number of different answers. Also in this paper, we cannot give you a complete or definite answer. Instead, we describe our experiences with various types of valves – experience that was built up during many years of conducting Stark deceleration experiments – but we leave it up to the reader to value our conclusions.

We describe our experiences with in particular the General Valve (GV), Jordan Valve (JV), and Nijmegen Pulsed Valve (NPV) as a source for Stark deceleration experiments. We study valve performance regarding opening time duration, optimal valve-to-skimmer distance, mean velocity, velocity spread, state purity, and relative intensity. We put particular emphasis on their suitability in crossed beam scattering experiments that employ a decelerator. Where possible, we support our experiences with measurements on the Stark deceleration of ND _3_ molecules, and with numerical simulations. In addition, we describe the procedures we have developed to calibrate valve performance, and to optimally load the beam into the decelerator.

This article is organized as follows. We first describe the three valves relevant to this study and discuss their opening mechanisms. This is followed by a description of the experimental set-up and procedures. In particular, we discuss the procedures to optimally load the beam pulse into the decelerator, and to experimentally characterize beam parameters such as mean velocity and opening time duration from measured time-of-flight profiles. We report on measurements of beams of ND _3_ that pass through a 2.6 meter-long Stark decelerator, from which we quantify the opening characteristics of the JV and NPV.

## The general valve, Jordan valve, and Nijmegen pulsed valve

We first very briefly describe the operation mechanisms of the GV, JV and NPV; for a more detailed and thorough description the reader is referred to the existing literature.

The commercially available GV (Parker Hannifin Corporation) is a magnetically activated solenoid valve. The valve opens by pulsing current through a solenoid, creating a magnetic field that lifts a plunger from the valve orifice. The opening time for a well-adjusted valve depends on the valve temperature, but is typically between 80 and 150 *μ*s (FWHM). The also commercially available JV (R.M. Jordan, Co.) is based on the original design by Gentry and Giese [32], and is operated by passing a high (3 - 4 kA) current in opposite directions through two metal strips. Due to the magnetic repulsion, the strips bend apart, opening the valve. This valve has a specified opening time of about 50 - 60 *μ*s (FWHM). Finally, the operation of the NPV is based on the Lorentz force on a current-carrying strip in a magnetic field [31]. A very light metal alloy strip is placed between two permanent magnets. When a current is pulsed through the strip, the Lorentz force lifts the strip from the valve orifice, opening the valve. Since the strip has a mass of only 23 mg, opening times of 20 *μ*s (FWHM) or shorter can be obtained.

A selection of the most important parameters of the valves is given in Table 1, summarizing operation mechanism, typical voltages and currents used to activate the valve, and typical opening times obtained. Table 1 also specifies the nozzle diameters of the valves that were available to us, and the typical pressures that were recorded in the source chamber under operating conditions. This pressure results from a combination of opening time, nozzle diameter, and backing pressure of the pulsed valve, and the density of the molecular beam formed. In all experiments described here, the molecular beam production parameters (such as backing pressure) for a given valve were also chosen such to produce an optimal molecular beam pulse, regarding intensity, velocity spread and rotational cooling. Gas mixtures of about 0.5 - 1 % ND _3_ in Ar, Kr or Xe were used. Typically, backing pressures of 1.0 bar and 3.3 bars were used for the NPV and JV, respectively.

**Table 1:** Three different molecular beam sources, and a selection of their characteristics

	Opening	Opening time	Operating	Operating	Nozzle	Pressure source
	mechanism	FWHM (*μ*s)	voltage (V)	current (A)	diameter (mm)	chamber (mbar)
General Valve	solenoid	80 - 150	300	1	1.0	4 · 10 ^−5^
Jordan Valve	current loop	50 - 60	25	4000	0.5	2 · 10 ^−5^
Nijmegen Pulsed Valve	Lorentz force	15 - 25	10 - 15	400 - 1000	0.5	2 · 10 ^−6^

Pressure source chamber refers to the typical pressure in the source chamber in our experiments under operating conditions (10 Hz)

## Experiment

**Fig. 1:**
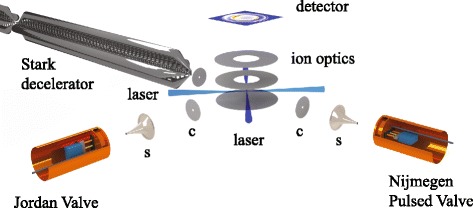
Schematic representation of the experimental set-up. A pulsed beam of ND _3_ or NO molecules seeded in Ar, Kr or Xe is produced by either a Jordan Valve or a Nijmegen Pulsed Valve and loaded into the Stark decelerator. After passing through this 2.6 meter-long Stark decelerator, of which only the last section is shown, the molecules are detected in the interaction region using Resonance Enhanced Multi Photon Ionization (REMPI). A one-color (2+1) or a two-color (1+1’) REMPI scheme is used to detect ND _3_ or NO, respectively. The microchannel plate detector can either be used to record the integral ion signal, or to record the velocity of the molecules using VMI. Molecular beams produced by a Jordan Valve and a Nijmegen Pulsed Valve are available at an angle of 90° and 180° with respect to the Stark decelerator, respectively. Both beams are collimated by a skimmer (s) and a collimator (c). These beams allow for scattering experiments, but in this study they are used to characterize the velocity distribution of the conventional beams

The experiments were performed in a molecular beam machine that is optimized for high-resolution crossed beam scattering experiments. It consists of a 2.6 meter-long Stark decelerator, two conventional molecular beams that are positioned at an angle of 90° and 180° with respect to the Stark decelerator’s axis, and a velocity map imaging (VMI) detector. This apparatus is schematically shown in Fig. 1, and has been described in detail elsewhere [33]. We here only describe the parts that are most relevant for the studies presented here.

The Stark decelerator is loaded with a molecular beam that is produced in a relatively large source chamber, that is pumped by a 1200 l/s turbo pump. The molecular beams formed are thus not limited by interactions with the vacuum chambers walls, or by limited pumping capacity. A 3 mm diameter commercially available skimmer (Beam Dynamics, model 2) is positioned between the source chamber and the Stark decelerator. A unique feature of our apparatus is that this skimmer is mounted on a movable gate valve [34], such that the source chamber can be vented without the need for breaking the vacuum in the remainder of the apparatus. This ensures the fast exchange of valves or adjustment of valve-to-skimmer distance.

Either a JV or a NPV is used to produce the molecular beam. Both valves are mounted in a cylindrical tube, which is inserted in a cylindrical donut-shaped bracket that is suspended inside the source chamber. This bracket is aligned with respect to the skimmer and axis of the Stark decelerator, and ensures that valves are positioned correctly and reproducibly. The distance between valve orifice and skimmer can be varied between 2 cm and 20 cm, and is adjusted by simply moving the tube inside the bracket.

In this work, mostly ND _3_ molecules are used, as this molecule is amenable to the Stark deceleration technique, and is a stable gas. Molecular beams of ND _3_ can thus be produced by simply seeding small amounts of ND _3_ in a carrier gas, without the need for production methods that can disturb the beam such as photodissociation or dissociation in a discharge. In a limited number of experiments that involve VMI, NO radicals are used. The quantum state for ND _3_ and NO that is selected by the Stark decelerator is the upper inversion level of the |*J*_*K*_〉=|1_1_〉 state, and the upper *Λ*-doublet level of the *X* ^2^ *Π*_1/2_,*J*=1/2 state, respectively.

The 2.6 meter-long Stark decelerator consists of 317 pairs of high-voltage electrodes that are placed horizontally and vertically in an alternating fashion [9]. The electrodes are spaced (center-to-center) 8.25 mm with respect to each other in the longitudinal direction, and they leave a 3 × 3 mm ^2^ opening for the beam to pass. All electrodes that are mounted in a certain direction are connected to each other such that the decelerator is operated with only four independent high voltage switches. The operation and characterization of a Stark decelerator has been described in detail before [35], and will not be repeated here. The Stark decelerator is operated in the *s*=3 mode [36] throughout, and a voltage difference of 30 kV is applied between opposing electrodes. A phase angle of *ϕ*_0_=0° is used to guide the beam through the decelerator at constant speed; deceleration occurs for *ϕ*_0_>0°.

The packet of molecules emerging from the decelerator is detected using Resonance Enhanced Multi Photon Ionization (REMPI) at a distance of 72 mm from the exit of the decelerator. For ND _3_, we use a one-color (2+1) REMPI scheme at a wavelength around 317 nm. A commercially available dye laser is used to generate light at the appropriate wavelength. Typically, the dye laser produces an energy of around 15 mJ per pulse in a 4 mm diameter spot, while the laser is focussed into the interaction region by a spherical lens with a focal length of 43 cm. For NO, we use a two-color (1+1’) REMPI scheme that allows for the ionization of NO molecules at the energetic threshold. This is essential for high-resolution measurements of the beam velocity distribution using velocity map imaging [25, 33].

A set of ion optics consisting of a repeller, an extractor and a grounded plate is used to accelerate the ions towards a microchannel plate detector (MCP) connected to a phosphor screen. The ions can be detected in two different ways. In the first, the output of the MCP detector is directly connected to a digital oscilloscope to record the integral ion signal. In this mode of detection, the voltages on repeller and extractor plates are chosen such that the ion signal is dispersed over a large area on the MCP to reduce possible detector saturation effects. This method is most suited to compare signal intensities. In the second, the repeller and extractor voltages are tuned to velocity mapping conditions, and two-dimensional images of the ion arrival positions are recorded using the phosphor screen and a CCD camera. This VMI mode of detection allows for direct measurements of beam speeds and velocity distributions [33, 37].

Additional molecular beam sources are installed at crossing angles of 90° and 180°. At 90° a JV is mounted, whereas a NPV is used at 180°. Both beam sources are separated from the interaction region by 2 mm diameter skimmers (Beam Dynamics, model 2) that are mounted at a distance of 87 mm from the interaction region. This configuration allows a direct comparison between the conventional beam pulses and the pulses that emerge from the decelerator.

### Procedures to optimally load the molecular beam into the decelerator

**Fig. 2:**
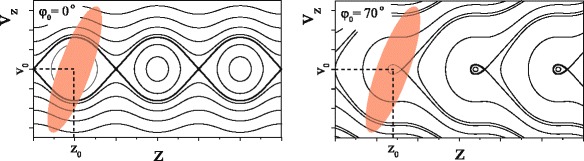
Schematic representation of the longitudinal phase-space acceptance of a Stark decelerator for guiding (*ϕ*_0_=0°, left) and deceleration (*ϕ*_0_=70°, right). The area in phase-space that is occupied by the molecular beam pulse at the entrance of the decelerator is schematically represented by the tilted red ellipse

To appreciate the various aspects that play a role when coupling the beam into the decelerator, we first discuss the operation principles of the Stark decelerator using the schematic representation in Fig. 2. It is instructive to use phase-space coordinates in the discussion, i.e., the position (*z*) and velocity (*v*_*z*_) of particles in the longitudinal direction. A proper discussion on the operation principles of Stark decelerators, and an introduction to the relevant terminology such as phase angle *ϕ*_0_ and synchronous molecule can be found elsewhere [28, 35]; we here restrict ourselves to the most basic concepts only.

The solid lines indicate the trajectories in phase-space that molecules will follow if the decelerator is operated using *ϕ*_0_=0° (left panel) or *ϕ*_0_=70° (right panel). Closed curves in the phase-space diagram correspond to bound orbits, i.e., molecules within the ’bucket’ or ’phase fish’ bound by the thick contour (called ’separatrix’) will oscillate around the synchronous molecule and are kept together [38]. These molecules are selected by the decelerator from the molecular beam pulse, and transferred to the final velocity at the end of the decelerator. This process occurs without losing any molecules in this bucket, and without the usual spreading of the molecules during the time they spend inside the decelerator. Note that since in a Stark decelerator all electrodes are coupled to each other, there is a series of buckets spaced by twice the distance between adjacent electrodes. For the decelerator used in this work, this distance is 16.5 mm.

The beam of molecules exiting the valve and passing the skimmer will occupy a certain area in phase-space. This area is schematically illustrated in Fig. 2 by the red ellipsoid. We here only discuss the situation in which the molecule of interest is a stable gas, i.e., the molecules are not produced at a well-defined time by a laser or discharge pulse. The exact shape of this area then strongly depends on the valve used: in the *z* direction, the width of the area is roughly determined by the opening time *τ* of the valve multiplied by the mean speed *v*_0_ of the beam. The longer the valve emits molecules, the wider the distribution will be. In the *v*_*z*_ direction, the width is determined by the velocity distribution of the molecules. Note that the ellipsoid is drawn slightly tilted to represent the evolution of the phase-space distribution in the free flight region between valve orifice and entrance of the Stark decelerator [35].

Optimal loading of the beam pulse into the decelerator is achieved if (i) the mean speed *v*_0_ of the beam coincides with the central speed of the first bucket, and (ii) the decelerator is switched on when the most intense part of the beam has reached the center position *z*_0_ of the first bucket. To experimentally find both parameters, and to select the appropriate timings and trigger pulses in the experiment, can be less straightforward than one may think. The largest uncertainty originates from the beam source itself. It is usually unknown at which time with respect to the valve trigger pulse the molecules are emitted from the valve. In addition, the final mean velocity of the beam is usually only reached at a significant distance from the nozzle (often only even after passage through the skimmer). In addition, the valve may produce a gradient in beam speeds over the valve temporal opening profile.

We therefore here describe a protocol to experimentally determine the correct timings, such that the pulse of molecules is optimally loaded into the first bucket. This protocol will also serve as an excellent proxy for the characteristics of the molecular beam pulse emitted from the valve. The protocol is further illustrated by experimental data on the guiding (*ϕ*_0_=0°) and deceleration (*ϕ*_0_=50°) of ND _3_ molecules seeded in Kr using an NPV. For this, the decelerator is programmed to select a packet of molecules with an initial velocity of 435 m/s, whereas the molecules emerge from the decelerator with a final velocity of 435 m/s and 313 m/s, respectively.

**Fig. 3:**
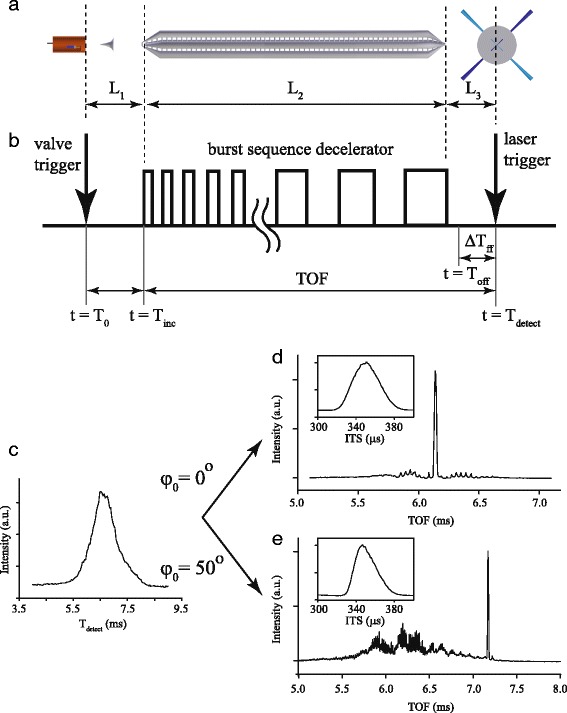
Schematic representation of the relevant distances in the experiment (**a**) together with a schematic representation of the trigger scheme (**b**) needed to synchronize the experiment. **c** Arrival time distribution of ND _3_ seeded in Kr at the detector, when the beam propagates in free flight from the source to the detector. Time-of-flight (TOF) distribution and Incouple Time Scan (ITS) when this beam is guided at constant speed (**d**) or decelerated (**e**). See text for details

The relevant distances in the experiment are defined in Fig. 3 a, together with a schematic representation of the trigger scheme in the experiment in panel *b*. The distance between valve orifice and Stark decelerator is denoted by *L*_1_, the decelerator itself has length *L*_2_, and the distance between exit of the decelerator and interaction region is given by *L*_3_. In the most basic trigger scheme, the experiment only involves three timings. We define the trigger pulse to open the valve as *t*=*T*_0_. The first high voltage pulse to the Stark decelerator is then applied at *t*=*T*_*inc*_. It is convenient to apply the burst sequence at the time when the synchronous molecule has reached the center of the first electrode pair. We refer to this time as the *incoupling time*. The Stark decelerator itself is operated by applying a burst of high voltage pulses to the electrodes through a programmable pulse generator. The burst itself is pre-calculated, and cannot (and should not!) be optimized during the experiment. Finally, the laser is fired at time *t*=*T*_*detect*_, leaving a time difference *Δ* *T*_*ff*_ between the laser trigger pulse and the time *T*_*off*_ at which the last high voltage pulse of the decelerator is switched off. During this time, the molecules propagate in free flight with the final velocity that was obtained in the last stage of the decelerator.

The first task is to obtain a rough estimate of the mean beam speed *v*_0_. This is most easily determined by measuring the arrival time distribution of the beam in free flight, i.e., no voltages are applied to the decelerator, and the signal on the detector is recorded as a function of *T*_*detect*_. This ensures that the beam is measured with no distortions originating from the switched electric fields. It is noted that if the length of the decelerator does not allow for such free flight measurements, a DC voltage of a few kV can be applied to the decelerator to raise signal levels. A time-of-flight (TOF) profile for ND _3_ seeded in Kr is shown in panel *c*. The beam arrives about 6.6 ms after *T*_0_, and the arrival time distribution has a width (FWHM) of about 12 %. From the mean arrival time, and the known dimensions of the experiment, a first rough estimate of the mean beam speed *v*_0_ is obtained.

The protocol starts with applying a time sequence to the decelerator based on *v*_0_ and *ϕ*_0_=0°, and by configuring the trigger scheme such that the laser trigger pulse is defined with respect to *T*_*inc*_, rather than *T*_0_ or *T*_*off*_. In the remainder of this paper, we therefore refer to TOF as the time difference between *T*_*detect*_ and *T*_*inc*_, i.e, the TOF is the time needed to propagate from the entrance of the Stark decelerator to the interaction region, *independent of the time spent in the source region*. The value for *Δ* *T*_*ff*_ is calculated from the final velocity of the packet – which is again *v*_0_ for *ϕ*_0_=0° – and the known distance *L*_3_^1^. The advantage of linking the laser trigger to *T*_*inc*_ is that now *T*_*inc*_ can be scanned with respect to *T*_0_. Referring back to Fig. 2, such a scan effectively moves the buckets along the *z*-axis. Since the laser trigger pulse was already synchronized to the burst sequence (and thus final velocity of the packet), a large signal should appear on the detector whenever the beam overlaps with the first bucket. We refer to such a scan as incouple time scan (ITS). Such an ITS for a time sequence with *v*_0_=435 m/s and *ϕ*_0_=0° is shown in the inset to panel *d*. The distribution is symmetric, and has a width (FWHM) of 33 *μ*s. This width results from a convolution of bucket size and the spatial extent of the beam at the entrance of the decelerator (see also Fig. 2). From this, we can estimate *τ*∼15−20*μ*s for the NPV.

The second step of the protocol is to select the optimal value of *T*_*inc*_ from the ITS, and to record a TOF as is shown in panel *d* by scanning *T*_*detect*_ with respect to *T*_*inc*_. If the rough estimate of *v*_0_ indeed represents the mean velocity of the beam, a symmetric TOF should result, featuring a central intense peak and a series of small wiggles on either side of the central peak. A detailed interpretation of this structure is given elsewhere [35]. If the TOF is not symmetric, i.e., there is substantially more signal intensity on one side of the central peak, the protocol must be repeated using a new value of *v*_0_ until a symmetric TOF is obtained. This procedure will result in the optimal selection of *v*_0_ and *T*_*inc*_.

In the third step of the protocol the molecules are decelerated by simply applying a burst sequence pertaining to *v*_0_ and *ϕ*_0_>0° to the decelerator. Again, *Δ* *T*_*ff*_ is calculated from the exit velocity and *L*_3_. To record an ITS, *T*_*detect*_ is fixed based on the calculated time the molecules spend inside the decelerator, and the calculated value for *Δ* *T*_*ff*_; to record a TOF, *T*_*detect*_ is scanned with respect to *T*_*inc*_. The resulting ITS and TOF for *ϕ*_0_=50° are shown in panel *e*. Clearly, a packet of molecules is selected from the molecular beam pulse, is decelerated, and arrives in the interaction region at later times. The ITS is slightly asymmetric and has a width of only 25 *μ*s, reflecting the asymmetry and reduced size of the bucket compared to *ϕ*_0_=0°. Note that the maximum in the ITS for *ϕ*_0_=0° and *ϕ*_0_=50° is found at almost the same time. This is the consequence of our choice to define *T*_*inc*_ as the time when the synchronous molecule has reached the first electrode pair. The different center positions *z*_0_ for buckets pertaining to different values of *ϕ*_0_ are thus incorporated in the decelerator burst sequence, and for a given value for *v*_0_ identical values for *T*_*inc*_ are used throughout.

### Influence of opening time on time-of-flight profiles

In the experimental TOF profiles as presented in panels *d* and *e*, the selected packet of molecules is well separated from the remainder of the molecular beam. This is even the case for the guiding sequence in panel *d* (*ϕ*_0_=0°), where the final velocity of the packet is identical to the mean velocity of the molecular beam pulse. This ’contrast’ is the result of the relatively small value for *τ* of the NPV.

**Fig. 4:**
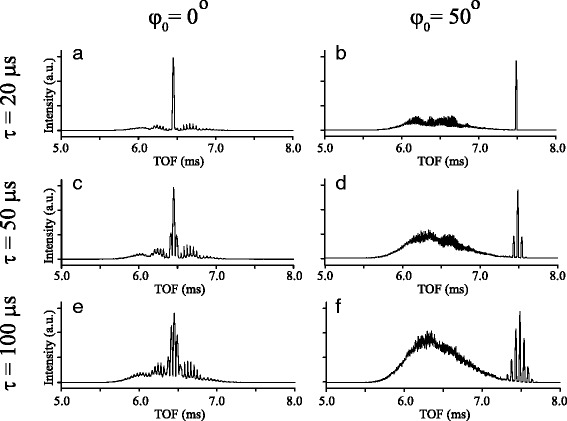
Simulated time-of-flight profiles for ND _3_ molecules exiting the Stark decelerator for guiding (*ϕ*_0_=0°, left column) and deceleration (*ϕ*_0_=50°, right column) when the opening time of the pulsed valve is 20 *μ*s (**a** and **b**), 50 *μ*s (**c** and **d**) or 100 *μ*s (**e** and **f**)

Indeed, an important criterium for the use of Stark-decelerated beams in crossed beam scattering experiments is the ability to produce a wide range of velocities with a TOF contrast that is similar to the ones presented in Fig. 3. Let’s therefore have a closer look at the influence of the valve opening time *τ* on the quality of the TOF profiles. Figure 4 shows TOF profiles that result from numerical trajectory simulations of different deceleration processes. These simulations pertain to the experimental conditions as present in the TOFs of Fig. 3, i.e., *v*_0_=435 m/s and *ϕ*_0_ is either 0° (left column) or 50° (right column). Three different valve opening times are used in the simulations: *τ*= 20 *μ*s (panels *a* and *b*), *τ*= 50 *μ*s (panels *c* and *d*), and *τ*= 100 *μ*s (panels *e* and *f*). These are representative values for *τ* for an NPV, JV, and GV, respectively.

Clearly, the best contrast in the TOFs is obtained for valves with the shortest opening times. For large values of *τ*, the spatial extent of the beam at the entrance of the Stark decelerator exceeds the size of the first bucket. Multiple buckets can be filled, resulting in multiple peaks in the TOF. The spatial extent of the beam scales with *v*_0_ *τ*, such that for *v*_0_= 435 m/s one expects to start filling the second bucket when *τ*>35*μ*s. This is indeed seen for *τ*= 50 *μ*s in Fig. 4, where the signature of the second bucket can be identified.

For *τ*= 100 *μ*s, multiple buckets are filled and the TOFs become progressively more congested. This has indeed been observed in Stark deceleration experiments that use a GV as beam source [39, 40]. Whereas for trapping experiments such a TOF does not necessarily impose a problem (as only a single bucket will be loaded into a trap), crossed beam scattering experiments may suffer significantly from the reduced contrast.

Based on these considerations, we consider the opening time of a GV too large for scattering experiments, and the use of this valve as a source for Stark deceleration experiments should be carefully considered. In addition, from extensive experience with various types of valves, the GV produces beams with rather low peak intensity. In the remainder of this paper, we therefore focus exclusively on the differences between the JV and NPV.

## Results and discussion

### Time-of-flight profiles using a Jordan valve and Nijmegen Pulsed Valve

**Fig. 5:**
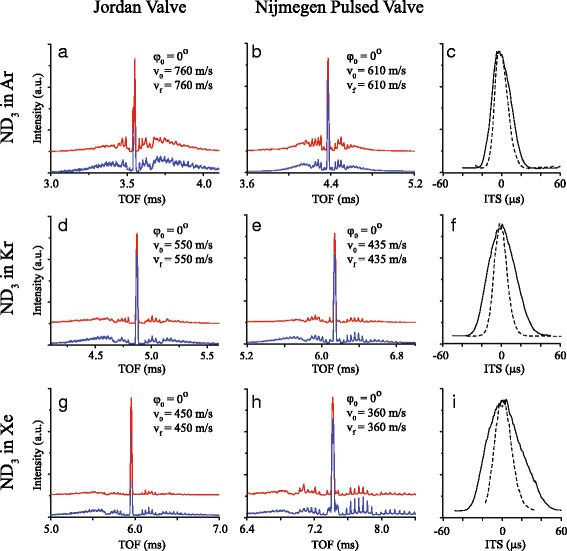
Experimental (red curves) and simulated (blue curves) TOF profiles for ND _3_ molecules exiting the Stark decelerator, when a Jordan Valve (left column) or a Nijmegen Pulsed Valve (middle column) is used. The Stark decelerator is operated to guide (*ϕ*_0_=0°) a packet of molecules through the decelerator at constant speed, and Ar (panel **a-c**), Kr (panel **d-f**), or Xe (panel **g-i**) is used as seed gas. The selected velocity from the molecular beam pulse, as well as the velocity with which the packet exits the decelerator, is indicated in each panel. The associated ITSs are shown in the right column (solid line: NPV, dashed line: JV)

We have systematically studied the performance of a JV and NPV as a source for Stark decelerators by recording series of TOFs following the beam loading protocol described above. Figure 5 shows a set of TOFs pertaining to guiding (*ϕ*_0_=0°) when the seed gases Ar, Kr and Xe are used. The results for a JV and NPV are shown in the left and middle columns, respectively, whereas the corresponding ITSs are shown in the right column. The peak positions of the ITSs are shifted with respect to *T*_0_ for ease of comparison. In all TOFs, an intense central peak is observed that contains the guided bunch of molecules. These peaks show excellent contrast with respect to the signal intensity on either side of this peak.

**Fig. 6:**
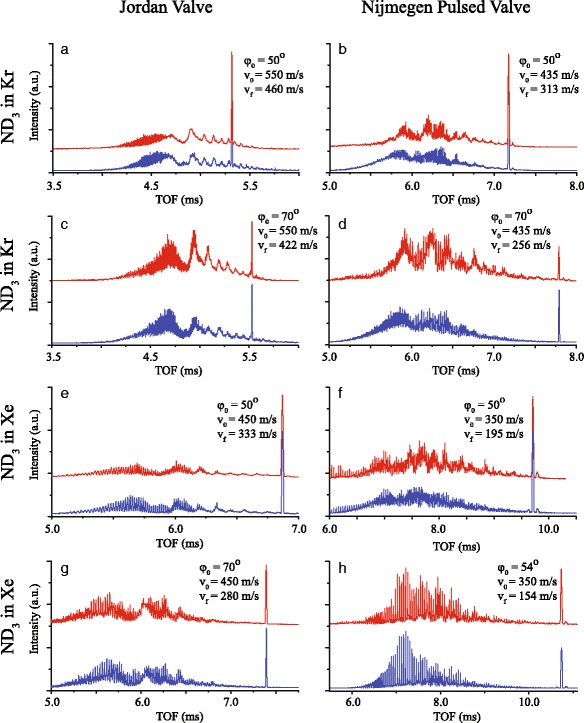
Experimental (red curves) and simulated (blue curves) TOF profiles for ND _3_ molecules exiting the Stark decelerator, when a Jordan Valve (left column) or a Nijmegen Pulsed Valve (right column) is used. The Stark decelerator is operated to decelerate (*ϕ*_0_>0°) a packet of molecules, and Kr (panel **a - d**) or Xe (panel **e - h**) is used as seed gas. The selected velocity from the molecular beam pulse, the phase angle *ϕ*_0_ that is used in the decelerator, as well as the velocity with which the packet exits the decelerator, is indicated in each panel. Peak intensities are normalized. The Jordan Valve is approximately three times less intense compared to the Nijmegen Pulsed Valve. Note that the Nijmegen Pulsed Valve allows for deceleration to lower final velocities for identical phase angles and carrier gases compared to the Jordan Valve

The TOFs pertaining to deceleration using Kr and Xe as a carrier gas, and using the phase angles 50° and 70°, are shown in Fig. 6. It is noted that when an NPV and Xe are used, a final velocity of 154 m/s is reached when *ϕ*_0_=54°. This is the lowest final velocity obtainable in the *s*=3 mode of operation [41], and deceleration to lower final velocities is beyond the scope of the studies presented here. In all TOFs, the decelerated bunch of molecules is separated from the remainder of the gas pulse, and its signature is clearly visible. Interesting broad and narrow oscillatory structures are visible in the TOFs of the non-decelerated parts of the beam. These structures have been observed and interpreted before in Stark-deceleration experiments using the OH radical [35, 36].

It is seen that the JV produces beam pulses with a significantly higher mean speed than the NPV: for Ar, Kr, and Xe mean velocities of 760 m/s, 550 m/s and 450 m/s are found for the JV, whereas these values amount to 610 m/s, 435 m/s and 360 m/s for the NPV. This is attributed to the high current pulse that is used to operate the JV, which significantly heats up the valve body and gas reservoir. The mean speeds that are found when a NPV is used are in better agreement with what one may expect from a room temperature expansion. Note that in panel (*a*), the slightly asymmetric TOF indicates that the selected beam velocity of 760 m/s appears slightly higher than the mean velocity of the beam.

For both guiding and deceleration, the experimentally obtained TOFs (red curves) are in excellent agreement with the results from numerical trajectory simulations (blue curves). For these simulations, the opening time duration *τ* of the valve is deduced from the ITS shown in Fig. 5, and amounts to 20 *μ*s and 10 *μ*s for the NPV and JV, respectively.

### Characterization of valve opening characteristics

From the contrast observed in the TOFs, we may thus conclude that both the JV and the NPV will fill only one bucket, and are well suited as a source for Stark decelerators in crossed beam scattering experiments. Referring back to the ITSs of Fig. 5, however, the value for *τ* that is found for the JV is much shorter than the expected 50 - 60 *μ*s from its specifications. For the NPV, in contrast, the value for *τ* closely matches the opening time duration that is expected for this valve.

This apparently surprising result can be explained by the opening mechanisms of both valves, and the operation principles of the Stark decelerator. The NPV is activated using only small voltages, and a moderate power of about 1 W is dissipated in the valve. This results in only a modest change of the beam’s mean speed during the opening of the valve, i.e., the beam that is formed when the valve just opens is about the same as the mean speed that is found at later times. This behavior is very different for the JV. Here, large currents are needed to open the valve, resulting in significant power dissipation (about 20 W) and a hot valve body. Moreover, as the valve opens, gas flows through the hot current conductors, locally cooling the conductors. As a result, the mean speed of the beam shows a strong gradient during the valve opening time [42]. When the valve just opens, the beam is very fast. The later the molecules leave the valve, the lower their mean speed will be. The slowest mean speeds are found when the valve is almost fully closed. Here, beam speeds that are expected for room temperature expansions are found [42].

**Fig. 7:**
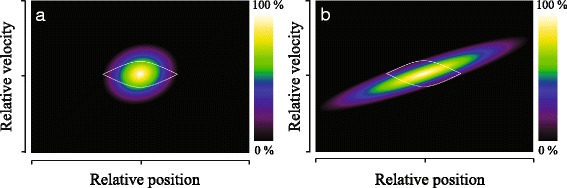
Longitudinal phase-space acceptance (white curve) of the Stark decelerator for *ϕ*_0_=0°, together with a schematic representation of the phase-space area of the molecular beam pulse at the entrance of the Stark decelerator for a Nijmegen Pulsed Valve (**a**) and a Jordan Valve (**b**)

This valve opening behavior has large consequences on the operation of Stark decelerators. Referring back to Fig. 2, the Stark decelerator selects the part of the molecular beam pulse that overlaps with the first bucket of the decelerator. In Fig. 7 the phase-space distribution of the molecular beam at the entrance of the decelerator is schematically shown for both valves. The relatively constant mean speed of the beam during the valve opening pulse for the NPV results in a phase-space distribution with a position spread that is approximately given by *v*_0_ *τ*. For Ar, Kr and Xe seeded beams this results in a spatial width of about 12, 9 and 7 mm, respectively, which is indeed smaller than the spatial dimensions of the decelerator bucket.

For the JV, however, the strong gradient in *v*_0_ results in a strongly tilted phase-space distribution. It is noted that this tilt is not the consequence of propagation of the beam in free flight to the entrance of the decelerator; the tilt is mainly due to the opening characteristics of the valve itself. The spatial distribution of the beam within the first bucket is therefore (much) narrower than the spatial distribution of the molecular beam itself, i.e., the temperature gradient during valve opening causes a strong correlation between speed and position of the molecules at the entrance of the decelerator. This correlation results in an *underfilling* of the first bucket at the selected mean velocity. This in turn causes a rather narrow temporal distribution when an ITS is measured. We thus conclude that the performance of a JV as a source for Stark decelerators is best described using an *effective* opening time *τ* that is much smaller than the actual opening duration of the valve.

This interpretation is supported by additional measurements on the beam speeds for both valves using VMI. For this, an additional JV or NPV is used without the decelerator such that the beam is detected directly behind the skimmer (see Fig. 1). In these measurements we use NO radicals instead of ND _3_, as NO can be ionized at threshold such that the mean speeds and velocity distributions can be measured accurately with VMI [25].

**Fig. 8:**
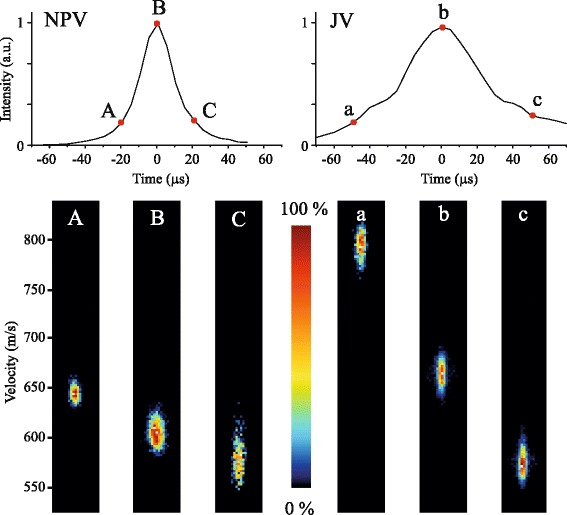
Arrival time distributions of NO radicals seeded in Ar, produced by an NPV (left) or JV (right) positioned at a distance of 87 mm from the interaction region. At selected arrival time positions, indicated by the letters A,B,C (NPV) or a,b,c (JV), the mean speed and velocity spreads of the packets are measured using velocity map imaging

In Fig. 8, the arrival time distributions are shown for beams of NO seeded in Ar when the NPV (left panel) or the JV (right panel) is used. Arrival time distributions with widths (FWHM) of 20 *μ*s and 45 *μ*s are found for the NPV and the JV, respectively. Clearly, the opening time of the NPV is much narrower compared to the JV. The mean speed of the molecules along the opening time duration of both valves is measured by recording the velocity distribution of the NO packets at the selected arrival times indicated in the figure. The resulting VMI images, referred to as beamspots, are shown for both valves in the lower part of Fig. 8.

The different behavior of both valves is immediately clear from the series of beamspots. The mean speed of the packet at the peak of the arrival time distribution is lower for the NPV (605 m/s) than for the JV (665 m/s). For both valves, the beginning part of the gas pulse is faster than the trailing part of the pulse. However, the difference in mean velocity over the temporal duration of the gas pulse is much smaller for the NPV compared to the JV. Similar behavior for both valves is found for beams of NO seeded in other rare gas atoms (data not shown).

### Intensity and state purity of the selected packet

Apart from the contrast obtained in TOF profiles, the intensity and state purity of the selected bunch of molecules is essential to the success of any collision experiment that is conducted after the deceleration process. We have found that the distance from the valve to the skimmer should be carefully chosen to obtain optimal beam densities, in accordance with observations by others [43]. It is noted that we here only describe the optimal distance for the skimmer that was available to our experiments; we explicitly emphasize that other skimmer shapes or opening diameters may not necessarily yield identical results ^2^.

In the experiment, the valve-to-skimmer distance is easily changed exploiting our skimmer gate-valve and valve mounting mechanism. The distance can thus be changed several times a day, and the effect of nozzle-skimmer distance is investigated under otherwise identical conditions. Peak densities are inferred from selecting the mean velocity of the molecular beam pulse, and by observing the peak signal intensity when this bunch is guided through the decelerator at *ϕ*_0_=0°. We have found that for the NPV, the nozzle-skimmer distance that yields highest beam densities amounts to about 18 cm, 13 cm and 11 cm when the carrier gases Ar, Kr and Xe are used, respectively. For the JV, similar values are found. This trend is rationalized from the overall decreasing peak densities when these gases are used; skimmer interference and clogging thus occurs at a larger valve-to-skimmer distance for the more intense Ar beams compared to less intense Xe beams.

An important question relates to the *relative* and *absolute* peak densities of the packets that emerge from the decelerator. A rule of thumb we find in our experiments is that beams produced using Ar yield about a factor 3 higher peak density compared to Kr seeded beams, which in turn yield about a factor 4 higher densities compared to Xe seeded beams. This trend and these ratios are found for both valves. The overall signal intensities, however, are significantly higher for the NPV compared to the JV: for optimal settings, the NPV yields about a factor 3 larger signal intensities throughout. This ratio appears rather constant for all gases.

It is notoriously difficult to measure the *absolute* density of the packet of molecules. The ND _3_ molecules are detected state-selectively with REMPI using a focused pulsed laser. Care was taken to saturate the optical transitions, but to limit saturation of the MCP detector and the detection system. Under these conditions, the number of ions detected is a good probe for the density of the molecular packet within the laser ionization volume. For the NPV with Ar as carrier gas, i.e., the largest signals available in the experiment, we measure about 20.000 ions per shot for the guided packet that exits the decelerator. From the calculated laser beam waist and Rayleigh range (the 5 mm diameter laser beam is focused using a spherical lens with focal length = 43 cm) we estimate the peak density of the packet to be a few 10^10^ cm ^−3^.

**Fig. 9:**
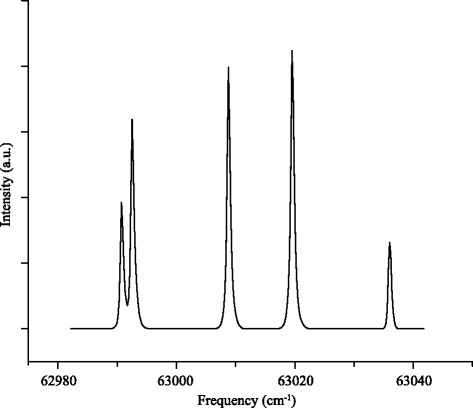
Experimental REMPI spectrum of ND _3_ molecules exiting the decelerator, probing exclusively the low-field seeking upper component of the inversion doublet for each rotational state. All lines in the spectrum originate from the upper inversion doublet component of the |*J*,*K*〉=|1,1〉 state. Population in other rotational states is negligible

Another important parameter is the quantum state purity of the packet of molecules emerging from the decelerator. Figure 9 shows a typical REMPI spectrum for ND _3_ molecules exiting the decelerator. The optical transition used probes exclusively the low-field seeking upper inversion level of each rotational state [17]. Each line in the spectrum originates from the |*J*,*K*〉=|1,1〉 rotational ground state of para-ND _3_. No signal at all is detected when probing the high-field seeking lower inversion levels (data not shown), although ionization must then occur in zero ion extraction field to eliminate parity mixing of both inversion doublets. Population in excited rotational states appears negligible, and we conservatively estimate that > 99.5 % of the molecules reside in this state.

## Conclusions

In this paper, we have described the requirements for molecular beam sources for Stark decelerators, in particular for the application of these decelerators in crossed beam scattering experiments. Apart from the straightforward desiderata such as beam intensity and rotational cooling, less straightforward requirements on the source exist. For instance, the selected bunch of molecules must exit the decelerator sufficiently decoupled from the remainder of the gas pulse, for a large range of final velocities, and this imposes stringent demands on the valve opening time duration.

The formation of the source molecular beam therefore deserves special attention in Stark deceleration experiments. Common wisdom in molecular beam production methods may not automatically apply to Stark deceleration experiments. The use of your favorite pulsed valve, although performing well in other molecular beam environments, should be carefully considered. For instance, in most deceleration experiments the carrier gases Kr and Xe must be used to reduce the initial speed of the beam. Under specific conditions, however, these gases are known to produce clusters in the supersonic expansion, and some valves may perform poorly with these carrier gases. The operation conditions of the valves in this study did not result in any significant clustering.

We have systematically investigated the suitability of a JV and NPV as a source for Stark decelerators, in particular for crossed beam scattering experiments. We have described a comprehensive protocol to determine the mean speed of the beam, and to optimally load the molecular beam pulse into the Stark decelerator. This protocol is also used to directly infer information on the spatial, velocity and temporal distribution of the gas pulse emitted from the valve. The protocol was applied to calibrate the performance of the JV and the NPV, using the guiding and deceleration of ND _3_ in a 2.6 meter-long Stark decelerator as a model system.

Both valves perform well, and are well suited to produce isolated packets of molecules within a large range of final velocities. The opening mechanism of the JV, however, requires high currents to open the valve. This leads to high mean beam speeds, and a peculiar correlation between velocity and position of molecules in the beam pulse. The former can be regarded as a true disadvantage, whereas the latter can be advantageous to select part of the beam pulse with the decelerator: a correlation between velocity and position is established at the entrance of the Stark decelerator, resulting in an *effective* valve opening time that is much shorter than the actual opening time. The NPV produces beams with a slower mean speed, with a more homogeneous velocity profile, and with temporal durations as short as 20 *μ*s. This opening time is sufficiently short to only fill a single bucket in a Stark decelerator. The peak number density of the molecular packet exiting the Stark decelerator appears a factor 3 higher for the NPV compared to the JV.

Using an NPV, the packets of ND _3_ molecules emerging from the decelerator can be tuned in velocity over a wide range, have a high peak density > 10 ^10^ cm ^−3^, and a state purity of > 99.5 %. From extensive experience with many different types of pulsed valves, the NPV at present appears to meet our special requirements best. In addition, the NPV is easily manufactured at relatively low cost. A disadvantage, however, is that the NPV can at present only be operated at room temperature, although there appears no inherent limitation to engineer a coolable version of the valve.

We emphasize that our findings do not qualify or disqualify pulsed valves not used in this study. The experiments presented here do not include the latest developed piezo activated valves [44] or solenoid valves, for instance. Ultimately, optimally engineered pulsed valves should basically all give the same results: the beam is determined by the very fast opening and closing of the orifice, through which the gas should flow into vacuum unrestrictedly. This requires a fast actuator, that displaces sufficiently far to ensure unrestricted flow. In this respect, the movement of a very light strip with the strong Lorentz force, as used in an NPV, meets this requirement at low levels of power dissipation.

As so often, the success of an experiment depends both on the availability of appropriate scientific apparatus, as well as on the careful selection of appropriate experimental methods and procedures. Whereas we cannot aid in the first, we hope that with this tutorial on the “nuts and bolts” of beam sources we can support early stage researchers with the second.

## Endnotes

^1^ We cannot stress enough the importance of an accurate calibration of the distance *L*_3_ in the apparatus. Without it, the protocol is much more difficult to implement!

^2^ We have found indications for skimmer interference effects, even for the largest nozzle-skimmer distances used.

## References

[1] Bethlem HL, Berden G, Meijer G (1999). Decelerating neutral dipolar molecules. Phys Rev Lett.

[2] Vanhaecke N, Meier U, Andrist M, Meier BH, Merkt F (2007). Multistage Zeeman deceleration of hydrogen atoms. Phys Rev A.

[3] Narevicius E, Libson A, Parthey CG, Chavez I, Narevicius J, Even U (2008). Stopping supersonic beams with a series of pulsed electromagnetic coils: an atomic coilgun. Phys Rev Lett.

[4] van de Meerakker SYT, Bethlem HL, Meijer G (2008). Taming molecular beams. Nat Phys.

[5] Bell MT, Softley TP (2009). Ultracold molecules and ultracold chemistry. Mol Phys.

[6] van de Meerakker SYT, Bethlem HL, Vanhaecke N, Meijer G (2012). Manipulation and control of molecular beams. Chem Rev.

[7] Narevicius E, Raizen MG (2012). Toward cold chemistry with magnetically decelerated supersonic beams. Chem Rev.

[8] Jankunas J, Osterwalder A (2015). Cold and controlled molecular beams: Production and applications. Ann Rev Phys Chem.

[9] Scharfenberg L, Haak H, Meijer G, van de Meerakker SYT (2009). Operation of a Stark decelerator with optimum acceptance. Phys Rev A.

[10] Meek SA, Bethlem HL, Conrad H, Meijer G (2008). Trapping molecules on a chip in traveling potential wells. Phys Rev Lett.

[11] Meek SA, Conrad H, Meijer G (2009). Trapping molecules on a chip. Science.

[12] Sawyer BC, Stuhl BK, Lev BL, Ye J, Hudson ER (2008). Mitigation of loss within a molecular Stark decelerator. Eur Phys J D.

[13] Osterwalder A, Meek SA, Hammer G, Haak H, Meijer G (2010). Deceleration of neutral molecules in macroscopic traveling traps. Phys Rev A.

[14] Trimeche A, Bera MN, Cromiéres JP, Robert J, Vanhaecke N (2011). Trapping of a supersonic beam in a traveling magnetic wave. Eur Phys J D.

[15] Lavert-Ofir E, Gersten S, Henson AB, Shani I, David L, Narevicius J (2011). A moving magnetic trap decelerator: a new source of cold atoms and molecules. New J Phys.

[16] Lavert-Ofir E, David L, Henson AB, Gersten S, Narevicius J, Narevicius E (2011). Stopping paramagnetic supersonic beams: the advantage of a co-moving magnetic trap decelerator. Phys Chem Chem Phys.

[17] van Veldhoven J, Küpper J, Bethlem HL, Sartakov B, van Roij AJA, Meijer G (2004). Decelerated molecular beams for high-resolution spectroscopy: The hyperfine structure of ^15^ND _3_. Eur Phys J D.

[18] Hudson ER, Lewandowski HJ, Sawyer BC, Ye J (2006). Cold molecule spectroscopy for constraining the evolution of the fine structure constant. Phys Rev Lett.

[19] Bethlem HL, Berden G, Crompvoets FMH, Jongma RT, van Roij AJA, Meijer G (2000). Electrostatic trapping of ammonia molecules. Nature.

[20] van de Meerakker SYT, Smeets PHM, Vanhaecke N, Jongma RT, Meijer G (2005). Deceleration and electrostatic trapping of OH radicals. Phys Rev Lett.

[21] Stuhl BK, Hummon MT, Yeo M, Quéméner G, Bohn JL, Ye J (2012). Evaporative cooling of the dipolar hydroxyl radical. Nature.

[22] Liu Y, Zhou S, Zhong W, Djuricanin P, Momose T (2015). One-dimensional confinement of magnetically decelerated supersonic beams of O _2_ molecules. Phys Rev A.

[23] Gilijamse JJ, Hoekstra S, van de Meerakker SYT, Groenenboom GC, Meijer G (2006). Near-threshold inelastic collisions using molecular beams with a tunable velocity. Science.

[24] Kirste M, Wang X, Schewe HC, Meijer G, Liu K, van der Avoird A (2012). Quantum-state resolved bimolecular collisions of velocity-controlled OH with NO radicals. Science.

[25] von Zastrow A, Onvlee J, Vogels SN, Groenenboom GC, van der Avoird A, van de Meerakker SYT (2014). State-resolved diffraction oscillations imaged for inelastic collisions of no radicals with He, Ne and Ar. Nat Chem.

[26] Vogels SN, Onvlee J, von Zastrow A, Groenenboom GC, van der Avoird A, van de Meerakker SYT (2014). High-resolution imaging of velocity-controlled molecular collisions using counterpropagating beams. Phys Rev Lett.

[27] Brouard M, Parker DH, van de Meerakker SYT (2014). Taming molecular collisions using electric and magnetic fields. Chem Soc Rev.

[28] Bethlem HL, Crompvoets FMH, Jongma RT, van de Meerakker SYT, Meijer G (2002). Deceleration and trapping of ammonia using time-varying electric fields. Phys Rev A.

[29] Irimia D, Dobrikov D, Kortekaas R, Voet H, van den Ende DA, Groen WA (2009). A short pulse (7 *μ*s fwhm) and high repetition rate (dc-5 khz) cantilever piezovalve for pulsed atomic and molecular beams. Rev Sci Instrum.

[30] Even U, Jortner J, Noy D, Lavie N, Cossart-Magos C (2000). Cooling of large molecules below 1 K and He clusters formation. J Chem Phys.

[31] Yan B, Claus PFH, van Oorschot BGM, Gerritsen L, Eppink ATJB, van de Meerakker SYT (2013). A new high intensity and short-pulse molecular beam valve. Rev Sci Instrum.

[32] Gentry WR, Giese CF (1978). Ten-microsecond pulsed molecular beam source and a fast ionization detector. Rev Sci Instrum.

[33] Onvlee J, Vogels SN, von Zastrow A, Parker DH, van de Meerakker SYT (2014). Molecular collisions coming into focus. Phys Chem Chem Phys.

[34] Küpper J, Haak H, Wohlfart K, Meijer G (2006). Compact in-place gate valve for molecular-beam experiments. Rev Sci Instrum.

[35] van de Meerakker SYT, Vanhaecke N, Meijer G (2006). Stark deceleration and trapping of OH radicals. Ann Rev Phys Chem.

[36] van de Meerakker SYT, Vanhaecke N, Bethlem HL, Meijer G (2005). Higher-order resonances in a Stark decelerator. Phys Rev A.

[37] Eppink ATJB, Parker DH (1997). Velocity map imaging of ions and electrons using electrostatic lenses: Application in photoelectron and photofragment ion imaging of molecular oxygen. Rev Sci Instrum.

[38] Bethlem HL, Berden G, van Roij AJA, Crompvoets FMH, Meijer G (2000). Trapping neutral molecules in a traveling potential well. Phys Rev Lett.

[39] Quintero-Perez M, Jansen P, Bethlem HL (2012). Velocity map imaging of a slow beam of ammonia molecules inside a quadrupole guide. Phys Chem Chem Phys.

[40] Jung S, Tiemann E, Lisdat C (2006). Cold atoms and molecules from fragmentation of decelerated SO _2_. Phys Rev A.

[41] Scharfenberg L, Kłos J, Dagdigian PJ, Alexander MH, Meijer G, van de Meerakker SYT (2010). State-to-state inelastic scattering of Stark-decelerated OH radicals with Ar atoms. Phys Chem Chem Phys.

[42] van de Meerakker SYT, Labazan I, Hoekstra S, Küpper J, Meijer G (2006). Production and deceleration of a pulsed beam of metastable NH (*a*^1^*Δ*) radicals. J Phys B: At Mol Opt Phys.

[43] Even U (2014). Pulsed supersonic beams from high pressure source: Simulation results and experimental measurements. Adv Chem.

[44] Abeysekera C, Joalland B, Shi Y, Kamasah A, Oldham JM, Suits AG (2014). Note: A short-pulse high-intensity molecular beam valve based on a piezoelectric stack actuator. Rev Sci Instrum.

